# BrainPhys Neuronal Media Support Physiological Function of Mitochondria in Mouse Primary Neuronal Cultures

**DOI:** 10.3389/fnmol.2022.837448

**Published:** 2022-06-14

**Authors:** Andreia Faria-Pereira, Mariana Temido-Ferreira, Vanessa A. Morais

**Affiliations:** Faculdade de Medicina, Instituto de Medicina Molecular João Lobo Antunes, Universidade de Lisboa, Lisbon, Portugal

**Keywords:** BrainPhys medium, Neurobasal medium, mitochondria, neuronal bioenergetics, primary mouse neurons

## Abstract

*In vitro* neuronal cultures are extensively used in the field of neurosciences as they represent an accessible experimental tool for neuronal genetic manipulation, time-lapse imaging, and drug screening. Optimizing the cultivation of rodent primary neuronal cultures led to the development of defined media that support the growth and maintenance of different neuronal types. Recently, a new neuronal medium, BrainPhys (BP), was formulated envisioning the mimicry of brain physiological conditions and suitability for cultured human iPSC-derived neurons and rat primary neurons. However, its advantages in mouse primary neuronal cultures and its effects in neuronal bioenergetics are yet to be demonstrated. In this study, we validated the beneficial use of BP in mouse primary neuronal cultures based on the observation that neuronal cultures in BP media showed enhanced ATP levels, which increased throughout neuronal maturation, a finding that correlates with higher mitochondrial activity and ATP production at later maturation stages, as well as an increased glycolysis response on mitochondrial inhibition and increased mitochondrial fuel flexibility. Taken together, our data demonstrate that BP medium promotes mitochondrial activity along with neuronal maturation of *in vitro* cultures.

## Introduction

*In vitro* primary neuronal cultures are of key importance to study the synaptic defects of animal models of a wide variety of neurological disorders. Additionally, with the increased knowledge of stem cell biology, the development of human-induced pluripotent stem cell-derived neuronal *in vitro* cultures has boosted discoveries on the on-set mechanisms of neurological diseases and generated new platforms for drug screenings (Cota-Coronado et al., 2020; Lamotte et al., 2021).

One crucial key aspect is that the environment provided by the culturing conditions of these neuronal cell populations mimics the physiological conditions that sustain neuronal viability, differentiation, and maturation. Different conditions have been used for neuronal cultures, with Neurobasal medium supplemented with B-27 (NB + b27) being one of the most commonly used conditions (Seibenhener and Wooten, 2012; Shabanipour et al., 2019; Cano-Jaimez et al., 2020). However, this medium contains a non-physiological concentration of glucose (25 mM, hyperglycemic levels), saturating levels of neuroactive amino acids such as glycine, glutamate, serine, aspartate, and inorganic salts, which leads to an impaired action potential generation and synaptic communication of these cultures (Bardy et al., 2015).

To improve these issues, a chemical-defined neuronal medium, BrainPhys (BP), was developed to enclose the brain and spinal fluid physiological concentrations of glucose, calcium, inorganic salts, and reactive amino acids, as well as, similar osmolarity (Bardy et al., 2015). This medium was shown to support action potential firing and excitatory and inhibitory neurotransmission in human iPSC and ESC-derived neurons. Also, BP was suitable for acute mouse brain slices and to maintain *in vitro* rat primary neuronal cultures harboring active synapses (Bardy et al., 2015). Additional reports have further supported the benefits of performing neuronal cultures in BP, such as Alzheimer’s β-amyloid-related studies (Satir et al., 2020) and live-imaging experiments (Zabolocki et al., 2020).

Brain and, in particular, neuronal metabolism have been shown to be of particular importance for proper neurotransmission and synaptic activity. Mitochondria are key metabolic regulators; therefore, a proper mitochondrial function has been shown to be crucial to sustaining neuronal viability and activity (Rangaraju et al., 2014; Pathak et al., 2015; Princz et al., 2018), with mitochondrial dysfunction triggering several neurological disorders (Baloh et al., 2007; Winklhofer and Haass, 2010; Cassereau et al., 2011; Wang et al., 2020). Therefore, the use of a non-physiological media to maintain *in vitro* neuronal cultures may mask molecular mechanisms linked to underlying bioenergetic deficits altered in pathological models, ultimately undermining the success of putative therapeutics.

BrainPhys has been formulated envisioning the mimicry of physiological concentrations of several substrates, namely, glucose (2.5 vs. 25 mM in NB medium). Glucose has been reported as one of the most relevant brain fuels used by mitochondria to produce energy (Gibbs et al., 1942; Sokoloff et al., 1977; Dienel, 2019). However, whether this physiologically formulated BP medium is beneficial for neuronal mitochondrial activity remains yet unknown.

To understand the effects of BP medium on neuronal bioenergetics, we assessed the expression of synaptic and neuronal markers in mouse primary neuronal cultures maintained in either NB or BP media along with neuronal maturation. Simultaneously, the bioenergetic capacity and flexibility of neuronal mitochondria maintained in these two media were determined. Our data reveal that mouse neurons cultured in BP medium present an increase in neural network extension accompanied by an increase in the expression of synaptic markers. Bioenergetically, mouse neurons cultured in BP medium are more reliant on mitochondrial activity for energy production and are more dependent on mitochondrial substrates.

## Materials and Methods

### Mouse Primary Neuronal Culture

Mice were obtained from the iMM| JLA Rodent Facility (Lisbon, Portugal), where they were housed in a temperature-controlled room at 20–24°C. All the procedures were approved by the Portuguese National Authority for Animal Health (DGAV), as well as by the institute’s animals’ wellbeing office (ORBEA-iMM). This study was carried out in compliance with the ARRIVE guidelines (Percie du Sert et al., 2020).

**FIGURE 1:**
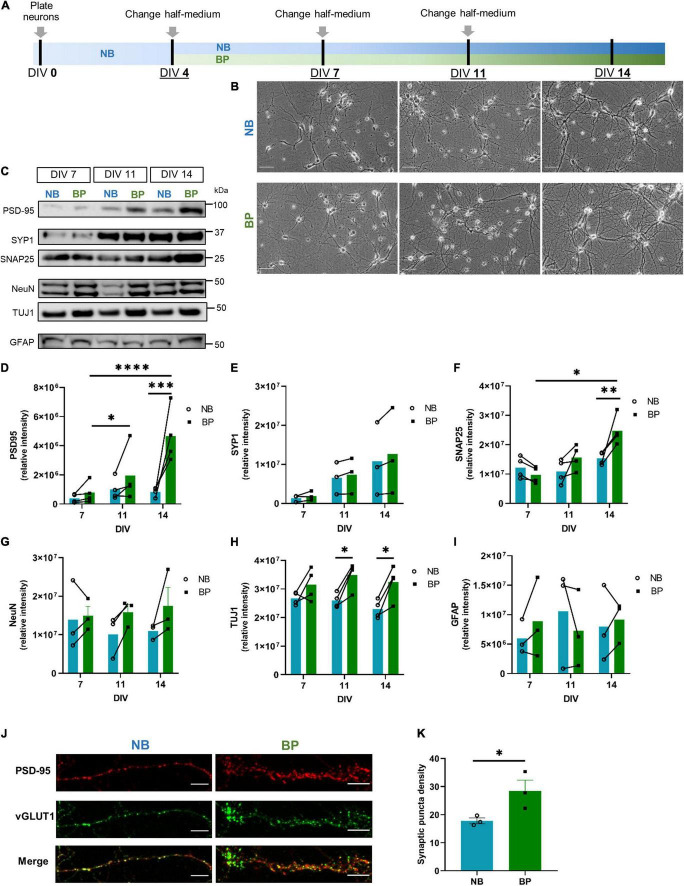
Denser neuronal networks and increased expression of synaptic markers in mouse neurons maintained in BrainPhys (BP) medium. **(A)** Schematic representation of mouse primary neuronal culture timeline employed to compare Neurobasal (NB) and BP media; **(B)** wide-field images of primary mouse neurons maintained in either NB or BP media at DIV7, DIV11, and DIV14 (scale bar = 100 μm); protein expression **(C)** and respective quantification of postsynaptic (PSD-95) **(D)**; presynaptic–Synaptophysin1 **(E)**, SNAP25 **(F)**; neuronal–NeuN **(G)**, TUJ1 **(H)**, and astrocytic–GFAP **(I)** markers in neurons maintained in NB or BP media and collected at DIV7, DIV11, and DIV14. **(J)** Expression of presynaptic (vGLUT1; green) and postsynaptic (PSD-95; red) markers in neurons maintained in NB and BP at DIV14. **(K)** Quantification of the area analyzed that is occupied by the synaptic puncta (scale bar = 10 μm). All graphs show means ± SEM determined by two-way ANOVA followed by Bonferroni’s multiple comparisons *post hoc* test **(D–I)** and unpaired student’s *t*-test **(K)**; * *p* < 0.05, **p < 0.01, ***p < 0.001, ****p < 0.0001 (*n* = 3–4 biological replicates).

Whole brains from E18 mouse embryos were used to establish mouse neuronal primary cultures. Neurons were cultures in NB medium (Gibco, 21103049) supplemented with 0.5 mM L-Glutamine (Gibco, 25030024), 20 U/ml Penicillin-Streptomycin, and B-27 (Gibco, 17504044), onward designated as “NB” medium, or in BP medium with SM-1 supplement (STEMCELL Technologies, #05792) and 20 U/ml Penicillin-Streptomycin, designated as “BP.” Half-medium changes were performed every 3–4 days, as depicted in Figure 1A. Specific details can be found in the Supplementary Section.

### Mitochondrial Function in Primary Neuronal Cultures

Mitochondrial function was evaluated by measuring the oxygen consumption rate (OCR) in the XF24 Extracellular Flux Analyzer (Seahorse Bioscience, Agilent, Santa Clara, United States). Analyses were performed on E18 mouse neurons at 10 and 15 days *in vitro* (DIV).

### Mitochondrial Membrane Potential Assessment

Neurons were plated on a 96-well tissue culture flat bottom plate and imaged at DIV9. To assess mitochondrial membrane potential, the cationic dye JC-1 (Tebu-Bio; 277MT09-10) was used.

### ATP Content Determination

Mouse neurons maintained in either NB or BP media until DIV7, 11, or 14 were collected in a guanidine-based extraction buffer as previously reported by Park et al. (2006). To determine adenosine triphosphate (ATP) content, a luciferase-based luminescent ATP determination assay was used according to the manufacturer’s protocol (Invitrogen).

### Cell Lysis and Immunoblot Analysis

Mouse neurons maintained in either NB or BP media at DIV7, 11, and 14 were lysed with RIPA buffer and further analyzed using the immunoblot technique. Primary and secondary antibodies used are listed in Supplementary Table I.

### Immunofluorescence Assay

Mouse neurons maintained in either NB or BP media at DIV7, 11, and 14 were fixed using 4% paraformaldehyde and further processed for immunofluorescence. Primary and secondary antibodies used are listed in Supplementary Table I. Images were captured using a Zeiss confocal microscope.

### Calcium Imaging

Calcium imaging was performed with Fura-2AM using neuronal cultures at DIV10 or 15 as described in the study by Ferreira et al. (2017). Image data were recorded and analyzed using the MetaFluor software (Universal Imaging).

### Statistical Analysis

Statistical analysis was performed using GraphPad Prism 8.0 (GraphPad Software, Inc., San Diego, United States). Data are presented as mean ± SEM. Differences were considered significant if the *p*-value was lower than 0.05.

## Results

### Mouse Neurons Maintained in BrainPhys Medium Show Increased Expression of Synaptic Markers

To determine the profile of mouse neuronal bioenergetics when cultured in BP or NB, mouse neurons were cultured for the first 4 days *in vitro* (DIV) in the NB medium. At DIV4, half-medium was replenished with fresh NB or BP medium. Half-medium changes were performed every 3–4 days (Figure 1A). Bright-field images show that *in vitro* maturation, characterized by an increased number and length of neurites, is evident in neurons maintained in both neuronal media (Figure 1B). It is also noticeable that, after DIV11 and more particularly at DIV14, the neuronal network of neurons maintained in the BP medium seems more vast and extended than of neurons maintained in the NB medium. To complement these observations, the expression of neuronal, astrocytic, and synaptic markers were assessed at DIV7, DIV11, and DIV14, respectively (Figures 1C–I). For the postsynaptic marker, postsynaptic density protein 95 (PSD-95), a discernible increase along maturation in both media is observed (Figures 1C,D), being significant for neurons maintained in the BP medium. However, neurons maintained in the BP medium present an overall tendency for higher expression levels of PSD-95 at all DIVs analyzed (DIV14 presents a statistical significance of *p* = 0.0006). Concerning the presynaptic markers, Synaptosphysin1 (SYP1), there is an increase in expression in both media from DIV7 to DIV14 (Figures 1C,E), but no differences between media are observed. Another presynaptic marker, SNAP25 (Figures 1C,F), revealed a tendency for lower expression in DIV7 neurons maintained in BP. However, along with maturation, this tendency overturns, and at DIV14, SNAP25 presents a significant increase of expression in neurons maintained in BP than in neurons maintained in NB (*p* = 0.007). To complement this increased expression of synaptic markers at DIV14 in neurons maintained in BP, the colocalization of vGLUT1 (presynaptic marker) and PSD-95 (postsynaptic marker), synaptic puncta, was assessed by immunofluorescence in NB and BP neurons at DIV14 (Figures 1J,K). From these results, neurons maintained in the BP medium showed a significant increase in synaptic puncta density (*p* = 0.04), in comparison with neurons maintained in NB. Concerning wide-ranging nuclear neuronal NeuN (Figures 1C,G) and TUJ1 (Figures 1C,H), their expression are maintained constant through neuronal maturation in both neuronal media levels, as expected. Nevertheless, for NeuN, there is a tendency, especially at DIV11 and DIV14, for higher expression in BP than in NB neurons, as previously observed (Jackson et al., 2018), and TUJ1 showed a significantly higher expression in neurons maintained in BP, at DIV11 and 14. Concerning the expression of the astrocytic marker GFAP (Figures 1C,I), overall a similar expression pattern was observed between NB and BP media (Figures 1C–I). Immunofluorescence data (Supplementary Figures 1a–c) corroborates that neurons in the BP medium display healthy neuronal morphologies and positively stained for standard neuronal markers, such as microtubule-associated protein TAU, TUJ1, and dendritic marker MAP2, which presents a wider expression at DIV14 in neurons maintained in BP than in NB, which correlated with the denser network observed in Figure 1B. Additionally, these cultures showed a clear enrichment in NeuN-positive cells, whereas GFAP-positive cells represented less than 6% in NB and BP media at DIVs 7-9 and 11-14 (Supplementary Figure 1d), similar to rat neuronal cultures (Sahu et al., 2019). The time-course of calcium imaging using fura 2-acetoxymethyl ester (Fura-2AM) was used to assess basal calcium levels and response to ionomycin, a Ca^2+^–ionophore in neurons maintained in the NB and BP media at DIV 10 (Supplementary Figure 1e) and 15 (Supplementary Figure 1f). We observed that neurons maintained in the BP medium have similar baseline intracellular Ca^2+^ levels (Supplementary Figure 1g), and the increase after ionomycin challenge was also similar (Supplementary Figures 1h,i) to neurons maintained in NB.

Therefore, as shown for human iPSC-derived neurons and rat primary neuronal cultures (Bardy et al., 2015; Satir et al., 2020), the BP medium is also suitable for mouse primary neuronal cultures.

### Mouse Neurons Maintained in BrainPhys Medium Show Increased ATP Content and Enhanced Expression of OxPhos Proteins

**FIGURE 2:**
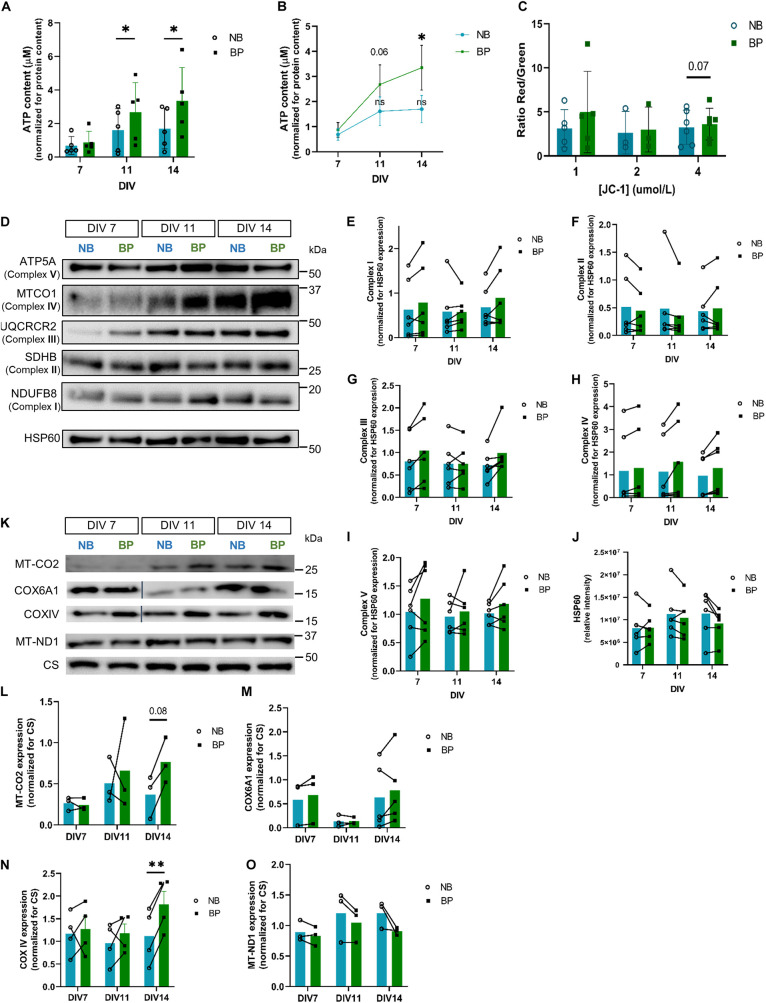
Mouse neurons maintained in BP medium present increased ATP content and increased expression of OxPhos proteins. **(A)** Comparison of ATP content between NB and BP neurons at DIV 7, 11, and 14 (*n* = 5–8 biological replicates) and **(B)** in NB and BP neurons throughout neuronal maturation (*n* = 5 biological replicates); **(C)** mitochondrial membrane potential measured by the quantification of red to green fluorescence ratio of DIV9 neurons maintained in NB or BP medium using different concentrations of JC-1 (*n* = 3–6 biological replicates); protein expression **(D)** and quantification of a subunit of each oxidative phosphorylation complex: NDUFB8 (Complex I) **(E)**, SDHB (Complex II) **(F)**, UQCRCR2 (Complex III) **(G)**, MT-CO1 (Complex IV) **(H)**, and ATP5A (Complex V) **(I)** in NB and BP neurons at DIV7, DIV11, and DIV14. Band intensities were normalized for mitochondrial matrix protein HSP60 expression (*n* = 6 biological replicates) **(J)**; protein expression **(K)**, and quantification **(L–O)** of Complex IV and I mitochondria I-encoded subunits—MT-CO2 and MT-ND1, respectively, and Complex IV nuclear-encoded subunits—COX6A1 and COXIV in NB and BP neurons at DIV 7, 11, and 14. Band intensities were normalized for Citrate Synthase (CS) expression (*n* = 3–4 biological replicates). All bar graphs show means ± SEM, determined by unpaired student’s *t*-test with Welch’s correction **(A,C)**, a two-way ANOVA followed by Bonferroni’s multiple comparisons *post hoc* test **(E–J,L–O)**, and a one-way ANOVA with Tukey’s correction **(B)**, *p < 0.05, ^**^p < 0.01.

Glucose is assumed to be the main source of energy in the brain (Gibbs et al., 1942; Sokoloff et al., 1977; Dienel, 2019), and neuron energy demands are known to be fulfilled by ATP derived mostly from oxidative phosphorylation (OxPhos), a process that occurs in mitochondria. Knowing that glucose concentration is 10-fold higher in the NB medium (25 mM) than in the BP medium (2.5 mM), a more physiologically relevant concentration for mammalian brains (Gruetter et al., 1992; Silver, 1994), it is expected that different glucose levels affect neuronal bioenergetics along with maturation. To address this, ATP content in neurons maintained in NB or BP medium was assessed (Figure 2A). Increased levels of ATP were observed in neuronal cultures in BP at DIV11 (*p* = 0.01) and DIV14 (*p* = 0.02). Interestingly, when analyzing ATP content throughout neuronal maturation (Figure 2B), there is a continuous increase of ATP levels in neurons maintained in BP, a finding that does not occur in neurons maintained in the NB medium. Assessing mitochondrial membrane potential using the ratiometric JC-1 probe, a significant difference between NB and BP neurons was not observed (Figure 2C), although neurons maintained in BP show a tendency for higher mitochondria membrane potential (*p* = 0.07).

The expression of OxPhos complex subunits has been shown to increase throughout neuronal maturation in cortical neurons (Agostini et al., 2016; Jády et al., 2016; Beckervordersandforth et al., 2017; Princz et al., 2018). When comparing OxPhos complex subunits, no significant expression differences are found for representative subunits of Complex I (Figures 2D,E), II (Figures 2D,F), III (Figures 2D,G), IV (Figures 2D,H), and V (Figures 2D,I) when compared with mitochondrial marker HSP60 (Figures 2D,J). Nevertheless, Complex IV subunit (MT-CO1) expression shows a tendency for increased expression in neurons maintained in BP, at DIV14. Since MT-CO1 is a mitochondrial-encoded protein, to distinguish if this tendency was derived from a general alteration in mitochondrial protein translation or from a generally increased expression of Complex IV subunits (either nuclear or mitochondrial-encoded) in neurons maintained in BP neurons, the expression of other mitochondrial-encoded OxPhos subunits (MT-ND1, Complex I subunit, MT-CO2, and Complex IV subunit) and nuclear-encoded Complex IV subunits, COX6a1 and COXIV, was assessed. At DIV14, neurons in the BP medium present a significantly higher expression of nuclear-encoded subunit COXIV (Figures 2K,N) (*p* = 0.001) and a tendency for higher expression of mitochondrial subunit MT-CO2 (Figures 2K,L) (*p* = 0.08). Complex IV nuclear-encoded subunit Cox6a1 was not found to be differentially expressed (Figures 2K,M). Curiously, a decreased expression at DIV11, which is then recovered at DIV14, is evident in neurons maintained in both media. In contrast, differences in the expression of the mitochondrial-encoded subunit of Complex I MT-ND1 were not observed when comparing neurons maintained in NB or BP (Figures 2K,O), suggesting that BP medium perhaps fosters increased Complex IV expression in mouse neurons.

### Mouse Neurons Maintained in BrainPhys Medium Show Increased OxPhos Activity

**FIGURE 3:**
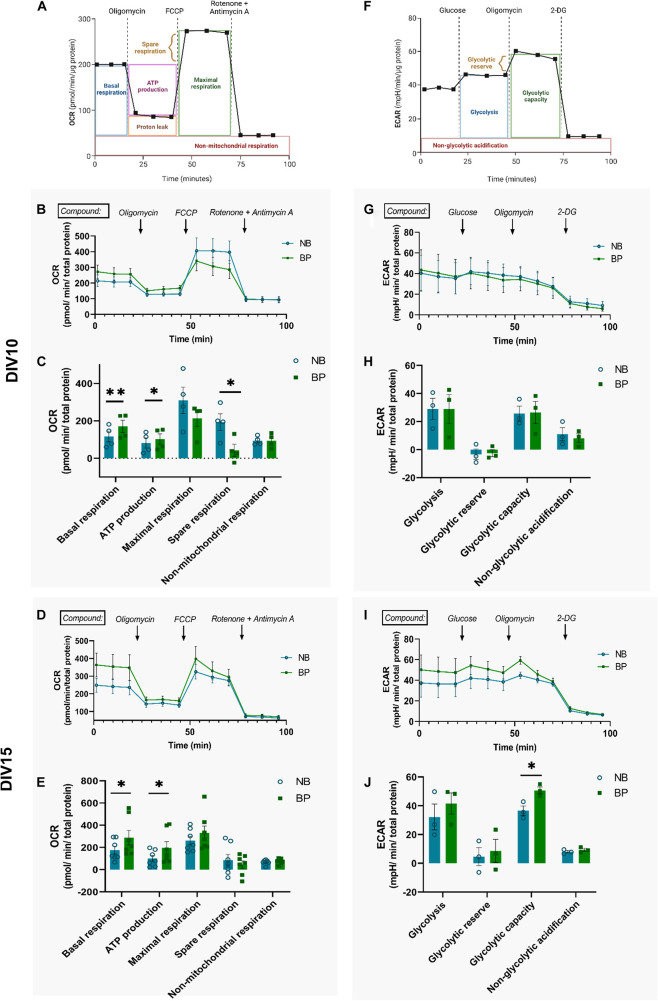
Mouse neurons maintained in BP medium present increased OxPhos activity. Representative graphs and measure parameters of oxygen consumption rate (OCR) **(A)** and extracellular acidification rate (ECAR) **(F)**. Time-course OCR graphs of NB and BP neurons at DIV10 **(B)** and DIV15 **(D)**. Quantification of basal respiration, ATP production on response to oligomycin, maximal and spare respiration on response to FCCP, and non-mitochondrial respiration on response to rotenone and antimycin A in NB and BP neurons at DIV10 **(C)** and DIV15 **(E)** (*n* = 4–7 biological experiments). Time-course ECAR graphs of NB and BP neurons at DIV10 **(G)** and DIV15 **(I)**. Quantification of NB and BP neurons showing glycolysis after glucose stimulus, glycolytic reserve, and capacity after inhibition of mitochondrial respiration through oligomycin and non-glycolytic acidification after 2-Deoxy-D-glucose (2-DG), an analogue of glucose that inhibits glycolysis at DIV10 **(H)** and DIV15 **(J)** (*n* = 3 biological experiments). All bar graphs show means ± SEM, determined using the two-sided unpaired student’s *t*-test, *p < 0.05; ^**^p < 0.01.

To assess the mitochondrial and glycolytic activity of neurons maintained in either NB or BP media, oxygen consumption (Figure 3A) and extracellular acidification (Figure 3F) rates were assessed at two different neuronal maturation points: DIV10 and DIV15. At DIV10 (Figures 3B,C), neurons maintained in the BP medium displayed increased basal mitochondrial respiration and ATP production capacity in comparison with neurons maintained in the NB medium. However, neurons maintained in the BP medium have a reduced ability to enhance OxPhos (spare respiration), on FCCP stimulus, when compared with neurons maintained in the NB medium (Figure 3C) (*p* = 0.04). Since neurons are cells highly reliant on OxPhos, it comes as no surprise that their mitochondrial respiration is very close to the maximum respiration (Hung and Calkins, 2016; Huuskonen et al., 2016; Lee et al., 2018; Faustini et al., 2019). Interestingly, later in neuronal maturation, at DIV15, neurons in BP also present a significant increase in mitochondrial respiration (*p* = 0.04), as well as ATP production (*p* = 0.02) (Figures 3D,E). These observations go in line with the previously observed increase in ATP content (Figures 2A,B). Although not significant, neurons maintained in the BP medium maintain a tendency for a reduced spare respiration capacity on FCCP stimulus. At both DIVs, differences in non-mitochondrial respiration were not observed between both media (Figures 3B–E). These data suggest that neurons maintained in BP are more reliant on mitochondrial activity than neurons maintained in NB. To assess if these differences in mitochondrial respiration might be due to a difference in mitochondrial mass, we quantified the mitochondrial density by immunofluorescence at DIV11 and DIV14 (Supplementary Figures 2a,b), and we found no differences in mitochondrial density between neurons maintained in NB and BP.

Although it is not the main ATP source in neurons, basal glycolysis rate and capacity were inferred from the measurement of extracellular acidification of the assay medium also at DIV10 and DIV15. At DIV10, all glycolysis activity parameters are similar between neurons independently of their culture conditions (Figures 3G,H). At DIV15, similarly to DIV10, glycolysis rate and glycolytic reserve are similar between neuronal media (Figures 3I,J). Interestingly, the capacity to shift to glycolysis when mitochondrial respiration is impaired (on oligomycin treatment) is significantly increased in neurons maintained in the BP than in the NB medium (*p* = 0.02) (Figure 3J). No differences were observed in non-glycolytic acidification of neurons maintained in either medium (Figures 3H,J).

### Mouse Neurons Maintained in BrainPhys Medium Show Higher Plasticity to Use Different Energy Fuel Sources

Generally, mitochondrial respiration relies on three main fuel sources, namely, glucose, fatty acids, and glutamine. Glucose is converted to pyruvate which can enter the mitochondria through the mitochondrial pyruvate carrier (MPC), where it is converted into acetyl-CoA and further used in the tricarboxylic acid (TCA) cycle. Long-chain fatty acids can also enter the mitochondria through the mitochondrial carnitine palmitoyltransferase 1 (CPT1) and through β-oxidation reactions originate acetyl-CoA, used in the TCA cycle. Glutamine, *via* glutaminase, can be converted into glutamate, which is then converted into α-ketoglutarate, which is also used in the TCA cycle. Applying specific inhibitors of transports/converters of these fuel pathways, we can individually inhibit each fuel pathway and assess the dependency of neuronal mitochondria respiration to each of these substrates (hereafter referred to as dependency). For this, we used the following inhibitors: UK-5099, an inhibitor of MPC; (+)-etomoxir, an inhibitor of CPT1a; and BPTES, an allosteric inhibitor of glutaminase. Additionally, it is also possible to inhibit two of the three main pathways and assess the capacity of neuronal mitochondria respiration to rely only on a specific fuel by inhibiting the other two main fuel pathways (hereafter referred to as capacity).

**FIGURE 4:**
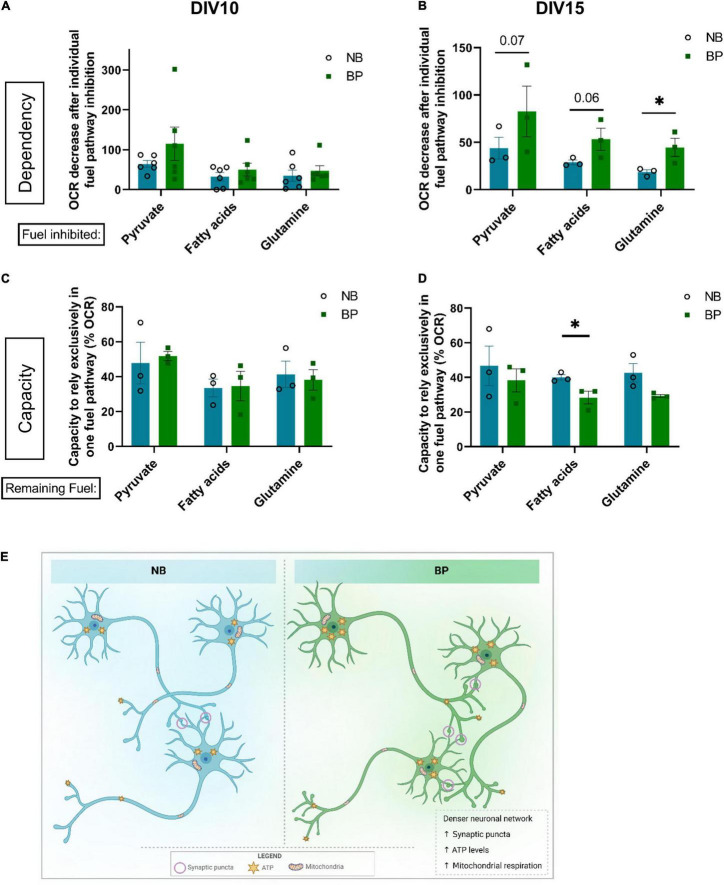
Mouse neurons maintained in BP medium show higher plasticity to use different energy fuel sources. OCR fraction quantification referred to the dependency of neurons maintained in NB or BP medium to rely on one of the three main fuel pathways: pyruvate, fatty acids, or glutamine on individual treatment with inhibitors of each fuel pathway: UK-5099, etomoxir, and BPTES, respectively. Measurements were performed at DIV10 **(A)** and DIV15 **(B)** (*n* = 3–6 biological experiments). Percentage of OCR regarding the capacity of neurons maintained in either NB or BP medium to rely exclusively on one of the three fuel pathways: pyruvate, fatty acids, or glutamine. Measurements were performed at DIV10 **(C)** and DIV15 **(D)** (*n* = 3 biological experiments). All graphs show means ± SEM, determined using the ratio paired student’s *t*-test, *p < 0.05. **(E)** In comparison with mouse primary neurons maintained in NB, neurons maintained in BP medium, after DIV4, showed a denser neuritic network correlated with increased expression of synaptic markers and increased synaptic puncta density. Bioenergetically, neurons maintained in the BP medium also presented increased ATP levels, which correlated with an increased mitochondrial respiration capacity.

To assess if a more physiological formulation of BP medium would impact the fuel preference and plasticity of neuronal mitochondria, the dependency and capacity to use the different fuels were assessed at different neuronal maturation points. At DIV10, independent of the media used, neurons are similarly affected if one of the three fuel pathways is inhibited (Figure 4A). Although not statistically significant, BP neurons seem to have a higher dependency on pyruvate oxidation into acetyl-CoA. The capacity of neurons in NB or BP medium to use only one of the main fuel sources (Figure 4C) is similar between media. Interestingly, at DIV15, there is a tendency for neurons maintained in the BP medium to be more dependent on oxidation of all the three main mitochondrial fuels (Figure 4B) with a statistical difference for glutamine dependency (*p* = 0.01). Additionally, it is noteworthy that, at both DIVs, neurons have a higher dependency on pyruvate oxidation for energy production independently of the culture media (Figures 4A,B).

Concerning the capacity to sustain mitochondrial respiration relying only on one of the fuels, no differences were found between media at DIV10 (Figure 4C). At DIV15, neurons maintained in BP show a tendency for lower respiration rates when only one mitochondrial fuel was available, with a significant difference when assessing fatty acid capacity (Figure 4D). These data suggest that neurons maintained in the BP medium present higher plasticity toward using a combination of different fuels.

## Discussion

This study sought to elucidate whether BP neuronal medium, previously formulated by Bardy et al. (2015), shown to be suitable for human iPSC-derived neurons, would be appropriate to culture primary mouse neurons, an *in vitro* model that is commonly used to assess the underlying molecular mechanism of neurodegenerative disorders (Slanzi et al., 2020). Thus, it is crucial that, in these *in vitro* primary neuronal culture models, physiological conditions are maintained as best as possible. As previously shown for human iPSC-derived neurons and rat primary neuronal cultures (Bardy et al., 2015; Jackson et al., 2018; Satir et al., 2020; Zabolocki et al., 2020), our findings demonstrate that neurons maintained in BP medium formed denser neuronal networks (Figure 4E), abundantly stained for neuronal and synaptic markers, and are functionally active, which is demonstrated by calcium imaging. Furthermore, the BP medium was formulated to mimic physiological brain conditions, with several substrate concentrations, namely, glucose, calcium, inorganic salts, and reactive amino acids, being adjusted to more physiological levels than in the commonly used NB medium (Bardy et al., 2015).

Moreover, this study aimed to unveil the impact of both neuronal media on neuronal bioenergetics. Our findings revealed that mouse neurons in the BP medium significantly increase ATP levels throughout maturation, and these levels are derived from mitochondrial activity (Figure 4E). These results are rather interesting when taking into account that even though the NB medium has a 10-fold increase of glucose when compared with BP, this concentration of glucose does not fuel the mitochondria, or glycolysis, to increase the ATP content of neurons cultured in the NB medium. This comes in line with the data demonstrating that hyperglycemic levels, as in patients with Type 2 Diabetes, impair mitochondrial homeostasis in different tissues (Santos et al., 2001; Mogensen et al., 2007; Raza et al., 2015; Czajka and Malik, 2016), as well as the usage of high glucose media impairs mitochondrial respiration and biogenesis in different cell lines and primary cells (Bayona-Bafaluy et al., 2021). This higher reliance on mitochondrial activity in BP neurons might correlate with higher synaptogenesis and synaptic maturation. It is known that mechanisms involved in neurotransmissions, such as synaptic transmission and propagation of action potentials, are some of the most energy-demanding processes in the brain (Attwell and Laughlin, 2001; Harris et al., 2012). Therefore, a neuronal medium that supports synaptic maturation and activity should also promote the reliance on mitochondrial activity, a feature that we clearly show in this study as a benefit of using the BP medium.

Interestingly, no differences in glycolysis were found in neurons maintained in either medium. However, at the latter maturation stages, BP neurons presented a higher capacity to increase glycolysis when mitochondrial respiration was impaired. Taking into account that glycolysis can be important to sustain energetic demands derived from synaptic activity (Rangaraju et al., 2014; Ashrafi et al., 2016; Jang et al., 2016), it is tempting to speculate that the increased neuronal network observed in BP neurons is also accompanied by an increased glycolytic capacity, which could be useful to promote and sustain a proper synaptic transmission in these *in vitro* models.

Glucose is the main energy fuel in the adult mammalian brain ranging from 1 mM to 2.5 mM in normoglycemic conditions and 2.7–4.5 mM in hyperglycemic conditions, which has 10-fold and 5-fold, respectively, lower glucose concentration than the one found in the widely used NB medium (25 mM) (Gruetter et al., 1992; Silver, 1994). Nevertheless, the usage of other fuel sources is also important, such as in brain development and growth (Jernberg et al., 2017; Sperringer et al., 2017). Other energy fuel sources are also very important to attenuate neurological disorders (Tieu et al., 2003; Yang et al., 2009; Cunnane et al., 2016), suggesting that neurons present certain plasticity to use other fuels besides glucose if required. Despite glucose concentration being one of the most sticking differences between NB and BP, other components have different concentrations between NB and BP medium. One such example is pyruvate, which is over two times more concentrated in the BP than in the NB medium. Interestingly, it has been shown in hippocampal neuronal cultures that, on supraphysiological glucose concentrations (30 mM), mitochondrial respiration was low; however, when acutely replaced by pyruvate, respiration increased (Pathak et al., 2015). A similar result was obtained when assessing mitochondrial respiration in synaptosomes (Choi et al., 2009). Therefore, it would not only be interesting to assess how supraphysiological glucose concentrations in BP medium, similar to NB medium, would impact the observed neuronal maturation and bioenergetics pattern but also how other compounds, such as glutamate and L-cysteine, which are increased in NB medium, have been shown to lead to excitotoxicity (Hogins et al., 2011), which in contrast has been shown to impact mitochondrial homeostasis (Wang et al., 2015).

In this study, when assessing the dependency of neuronal mitochondria on each individual fuel, neurons in the BP medium are more affected by inhibition of each individual fuel in the latter maturation stages than NB neurons, indicating that these neurons present higher fuel flexibility.

Additionally, as in early cortical neuronal maturation, where glycolytic intermediates, glutamate, citrate, and long-chain fatty acids were found to be increased through early neuronal maturation (Agostini et al., 2016), it would be interesting to assess the metabolic profile of neurons maintained in BP, in comparison with those in NB medium.

Worth mentioning is the fact that some glial cells are present in these cultures (GFAP-positive cells), which have been shown to increase long-term neuronal differentiation using the BP medium (Satir et al., 2020). However, in the time points reported in this study, an increased frequency of glial cells was not observed in mouse primary cultures in BP medium in comparison with NB medium cultures. Even though knowing that BP medium mimics the brain’s physiological environment, it should also promote the efficient development and maturation of glial cells; therefore, it would be very pertinent to study and characterize the effects of BP medium in the establishment and maturation of not only primary glial cultures but also with mixed neuronal-glial cultures (Chen et al., 2013).

A significant portion of studies using *in vitro* neuronal cultures aims at studying neuropathological defects mainly arising from neurodegenerative disorders, where one of the first pathological hallmarks is synaptic degeneration, accompanied by energy deficits. Therefore, significant impairments of genetic and pharmacological disease models may be camouflaged by the use of culture conditions that do not sustain a proper physiological mitochondrial activity crucial for synaptic activity and plasticity. Future studies using BP medium are necessary to unravel putative molecular, morphological, metabolic, and synaptic defects masked in disease and therapeutic models of neurological disorders.

Our results reveal the adequacy of the BP medium to generate robust neuronal networks of mouse primary neurons and, more importantly, unravel the novel aspect that neurons maintained in the BP medium are more reliant on mitochondrial activity, as it occurs in physiological conditions, than cultures with commonly used NB medium. Therefore, BP is a more physiologically suitable and bioenergetically favorable media in which mouse primary neuronal cultures should be performed and studied.

## Data Availability Statement

The raw data supporting the conclusions of this article will be made available by the authors, without undue reservation.

## Ethics Statement

The animal study was reviewed and approved by the Portuguese National Authority for Animal Health (DGAV), as well as by the institute’s animals’ well-being office (ORBEA-iMM).

## Author Contributions

AF-P and MT-F performed and analyzed the calcium experiments. AF-P performed the remaining experiments. AF-P and VM analyzed the data, designed the research, and wrote the manuscript. All authors have read and agreed to the published version of the manuscript.

## Conflict of Interest

The authors declare that the research was conducted in the absence of any commercial or financial relationships that could be construed as a potential conflict of interest.

## Publisher’s Note

All claims expressed in this article are solely those of the authors and do not necessarily represent those of their affiliated organizations, or those of the publisher, the editors and the reviewers. Any product that may be evaluated in this article, or claim that may be made by its manufacturer, is not guaranteed or endorsed by the publisher.
