# Supplementary material for: BrainPhys Neuronal Media Support Physiological Function of Mitochondria in Mouse Primary Neuronal Cultures
Source: Front Mol Neurosci. 2022 Jun 14;15:837448. doi: 10.3389/fnmol.2022.837448 (PMC9239074; doi:10.3389/fnmol.2022.837448)
Supplement: Supplementary file 1 [file Data_Sheet_1.docx]

Supplementary Material

**TITLE**: BrainPhys neuronal media supports physiological function of mitochondria in mouse pimary neuronal cultures

# Supplementary Material

Mouse primary neuronal culture

Whole-brains from E18 mouse embryos were collected in HBSS supplemented with HEPES 1M (HBSS-HEPES). Meninges were removed, and brains were homogenised and incubated with 0.25% trypsin and 10µl/ml DNaseI for 15min 37ºC. Tissue was washed with 10% Horse serum (HS) in HBSS-HEPES and centrifuged 1200 xg for 10min at RT. Supernatant was discarded and pellet was washed two more times with HBSS-HEPES. Pellet was resuspended in 10% HS in MEM, 0.6% Glucose and 100U/ml Penicillin-Streptomycin; and strained using a 70µm cell strainer. Cell viability was assessed using trypan blue. The desired amounts of cells (5x10^5^ cells/well - 6 well plate and 35mm glass bottom Petri dishes; 1x10^5^ cells/well - 24 well plate; 6x10^4^ cells/well - 96 well plate and Seahorse plate) were placed in 0.01mg/ml Poly-D-Lysine hydrobromide coated tissue culture plates. After 4h, medium was replaced with Neurobasal medium (Gibco, 21103049) supplemented 0.5mM L-Glutamine (Gibco, 25030024), 20U/ml Penicillin-Streptomycin and B-27 (Gibco, 17504044), onwards designated as “NB” medium. After 4 days, half-medium was changed with fresh medium: either with “NB” or with BrainPhys with SM-1 supplement (STEMCELL technologies, #05792) and 20U/ml Penicillin-Streptomycin, designated as “BP”. Recommended by the vendor and performed in other studies (González-Sánchez et al., 2017; Hangen et al., 2018; Jackson et al., 2018), half-medium changes were performed every 3-4 days, as depicted in Figure 1A.

Mitochondrial function in primary neuronal cultures

To assess overall mitochondrial activity, at the respective DIVs, neurons were placed in XF base medium supplemented with 10mM glucose, 2mM L-glutamine and 1mM sodium pyruvate, pH7.4, and incubated at 37ºC in the absence of CO2 for 30min. OCR measurements were performed over 3min after a 3min mix and 2min wait periods. Three measurements were collected for basal respiration, followed by three measurements after addition of 1.25µM Oligomycin, followed by three measurements after addition of 0.5µM FCCP (Carbonyl cyanide-4-(trifluoromethoxy) phenylhydrazone), followed by three measurements after addition of 1µM Antimycin A and 1μM rotenone, which represent the non-mitochondrial respiration. Basal respiration was calculated by the average of the first three measurements and substrate by the non-mitochondrial respiration. ATP production was calculated subtracting the OCR after oligomycin injection to the basal respiration. Maximal respiration was calculated by subtracting the non-mitochondrial respiration to the average of three measurements after FCCP injection. And spare respiration was calculated by subtracting the basal respiration to the maximal respiration.To assess overall glycolytic activity and capacity, at the respective DIVs, the same procedure was performed, however, XF base medium supplemented with 2mM L-glutamine was used. ECAR (Extracellular acidification rate) measurements were performed over 3min after a 3min mix and 2min wait periods. Three measurements were collected for basal glycolysis, followed by three measurements after addition of a saturating concentration of 25mM glucose, followed by three measurements after addition of 1.25µM Oligomycin and a final injection of 50mM 2-DG (2-deoxy-glucose),which represent the non-glycolytic acidification. Glycolysis was calculated by subtracting the non-glycolytic acidification to the ECAR average after glucose injection. Glycolytic capacity was calculated by subtracting the non-glycolytic acidification to the maximum rate measurement after oligomycin injection. Glycolytic reserve was calculated by subtracting the glycolysis to the glycolytic capacity.

To assess the bioenergetic fuel preference and dependency at the respective DIVs, OCR measurements were performed as previously described. For the dependency assay, three measurements were collected for basal respiration, followed by five measurements after addition of one of the three fuel inhibitors: 8µM Etomoxir, 4µM BPTES and 6µM UK-5099. This was followed by five measurements after addition of the two inhibitors that were not applied in the previous measurements at the designated concentrations. Three final measurements were performed after addition of 1µM Antimycin A and 1µM rotenone. The dependency was calculated by directly subtracting the basal (initial) OCR by the OCR at the end of the first inhibitor injections: basal OCR – OCR after the first inhibitor, similar to ATP production when assessing mitochondrial activity. For the capacity assay, similar layout was applied: basal respiration was measured followed by five measurements after addition of two of the three fuel inhibitors; followed by five measurements after addition of the remaining inhibitor and finalized by three measurements after addition of 1µM Antimycin A and 1µM rotenone. The capacity was calculated as: (basal OCR – OCR after the last inhibitor) – (basal OCR – OCR after the first two inhibitors), divided by the (basal OCR – OCR after the last inhibitor) and multiplied by 100 and expressed as percentage.

Mitochondrial membrane potential assessment

For uncoupling control, 100µM FCCP or EtOH was added and plate was incubated for 30min at 37ºC. JC-1 (Tebu-Bio; 277MT09-10) was added at the final concentrations of 1, 2 or 4µmol/L, and incubated for 30min at 37ºC. After incubation, JC-1 solution was replaced with 100µl HBSS-HEPES. Fluorescence intensity was measured (Tecan Infinite M200 Plate Reader) using the following Excitation/Emission wavelengths: 485nm/535nm for JC-1 monomers; 535nm/595nm for JC-1 aggregates. Values were plotted as a ratio of red to green fluorescence intensity.

ATP content determination

Before medium change, neurons were collected and lysed in a guanidine-based extraction buffer (6M Guanidine/HCl; 100mM Tris/HCl pH7.8; 4mM EDTA) as previously reported in (Park et al., 2006) After centrifugation, 5µg of each neuronal extracts were used to determine ATP content using a luciferase-based luminescent ATP Determination assay according to the manufacturer’s protocol (Invitrogen). Luminescence was measured on a plate reader (Synergy HTX Multi-Mode Reader, BioTek) and ATP amounts were determined in comparison to an ATP standard curve.

Cell Lysis and Immunoblot analysis

Lysates were performed using RIPA buffer (50mM Tris/HCl, pH8.0, 1mM EDTA, 1% Triton X-100, 0.5% sodium deoxycholate, 0.1% SDS, 150mM NaCl) supplemented with Protease inhibitors for 1h at 4ºC. After lysis, supernatant was collected and protein concentration was quantified using Pierce™ BCA Protein Assay Kit.

For immunoblot analysis, equal amount of protein extracts (10-40 μg/well) from both NB and BP were separated by SDS-PAGE (Invitrogen) in the appropriate running buffer (MOPS or MES) and transferred onto 0.2µm nitrocellulose membranes for 1h at 30V. Membranes were blocked with 5% low-fat milk powder in TBS-T (20mM Tris/HCl pH7.5, 150mM NaCl, 0.5% Tween-20) for 1h. Primary antibody incubations were performed overnight at 4ºC, and dilutions used are listed in Supplementary Table 1. Washes were performed for 15min at RT in TBS-T. The following secondary antibodies were used: Horseradish peroxidase (HRP) conjugated anti-rabbit and anti-mouse (Bio-Rad) at 1:10,000. Detection was done using the chemiluminescent ECL-Plus detection kit (Amersham) on a digital Amersham Imager 680 (GE Healthcare). Unsaturated immunoblotbands relative intensity was determined by densiometry measurements using Image Lite Studio 5.2 software (LI-COR Biosciences, Nebraska USA).

Immunofluorescence assay

Neurons were fixed in 4% paraformaldehyde in PBS+/+ (0.33mM MgCl2.6H2O, 0.9mM CaCl2.2H2O) for 20min at RT, and permeabilized with 0.1% Triton X-100 in PBS for 10min at RT. Blocking was performed for 1h at RT with blocking buffer (0.2% tissue culture grade gelatin, 2% FBS, 2% BSA, 0.3% Triton X-100 in PBS) supplemented with 5% goat serum followed by primary antibody incubation overnight at 4ºC. Dilutions used are listed in Supplementary Table I. Incubation with secondary antibodies conjugated with Alexa-488 and Alexa-568 (Invitrogen) were performed for 2h at RT using a 1:500 dilution. Coverslips were mounted in Mowiol (48 mg/ml Mowiol, 120 mg/ml glycerol in 48mM Tris/HCl, pH8.5). Images were captured on a Zeiss LSM 880 or a Zeiss LSM 710 confocal microscope with 63x oil objective, numerical aperture (NA) 1.4.

Mitochondrial density analysis was performed using the MiNA toolset (REF. Valente etal., 2017) to define the mitochondrial footprint, which was normalized by the TUJ1 signal area and presented as the percentage of mitochondrial density.

**Supplementary Table I**: Primary Antibodies used in this study

| **Antibody** | **Source** | **Catalogue number** | **Dilution** |
| --- | --- | --- | --- |
| Anti-PSD95 | Enzo Life Sciences | ADI-VAM-PS002-E | 1:1,000a; 1:100b |
| Anti-GFAP | Sigma-Aldrich | G9269 | 1:500a; 1:100b |
| Anti-Synaptophysin1 | Synaptic Systems | 101011 | 1:1,000a |
| Anti-TUJ1 | Biolegend | 802001 | 1:1,000a;1:100b |
| Anti-SNAP25 | Sigma-Aldrich | S9684 | 1:10,000a |
| Anti-NeuN | Cell Signaling Technology | #12943 | 1:1000a;1:400b |
| Anti-MAP2 | Sigma-Aldrich | M9942 | 1:200b |
| Anti-TAU | SynapticSystems | 314002 | 1:200b |
| Anti-vGLUT1 | SynapticSystems | 135303 | 1:100b |
| Anti-HSP60 | BD Biosciences | 611563 | 1:5,000a; 1:200b |
| anti-OXPHOS cocktail | Abcam | ab110413 | 1:2,000a |
| Anti-MT-CO2 | Abcam | ab198286 | 1:100a |
| Anti-MT-ND1 | Abcam | ab222892 | 1:500a |
| Anti-Cox6a1 | Abcam | ab110265 | 1:100a |
| Anti-COXIV | GeneTex | GTX114330 | 1:500a |

a - used for Immunoblot; b - used for Immunofluorescence

Synaptic puncta density analysis

Stacks of 5–8 images taken at 0.48 µm intervals were acquired with a 63X oil immersion objective (Zeiss) on a Zeiss LSM 710 confocal microscope. Briefly, each channel for each image was thresholded and several regions of interest (ROIs) were defined to include only signal from neurites, whose area was also measured. The synaptic puncta density was calculated as the area of the colocalized puncta normalized to the area of the analysed ROI, in order to account for neurites with larger diameters.

Calcium imaging

Using the procedure described in (Ferreira et al., 2017) with minor modifications, DIV 10 or DIV 15 neurons were loaded with 5µM Fura-2AM in a calcium physiological solution and incubated at 37°C, 5% CO_2_ for 45min. Cells were then placed on an inverted microscope with heated chamber with epifluorescent optics and equipped with a high speed multiple excitation fluorimetric system (Lambda DG4 with a 175W Xenon arc lamp). Data was recorded by a CDD camera. Fura-2AM loaded neurons were sequentially excited both at 340nm and 380nm, for 250ms at each wavelength, and the emission fluorescence was recorded at 510nm. Experiments were performed on cells with a baseline fluorescence ratio around 0.5, which corresponds approximately to a [Ca2+]i of about 100nM, considered the normal [Ca^2+]^I (Knot et al., 2005; Barhoumi et al., 2010; Ferreira et al., 2017). Cells with a baseline fluorescence ratio above 1 were discarded from the experiment. As a control, all cells were challenged with 2µM ionomycin (an effective Ca^2+^ ionophore) at the end of the experiment and only those that responded, confirming neuronal viability, were included in the analysis. Image data were recorded and analysed using the MetaFluor software (Universal Imaging).

Statistical analysis

Each set of data values were subjected to normality tests (Shapiro-Wilk test and Kolmogorov-Smirnov test and assessment of Q-Q plots). If data groups assumed a gaussian (normal) distribution, parametric tests were applied otherwise non-parametric tests were used. Statistical details of experiments, including the statistical tests used and the number of biological replicates (n), are noted in figure legends. Data are presented as mean ± SEM. Differences were considered significant if p-value was lower than 0.05.

# Supplementary Data


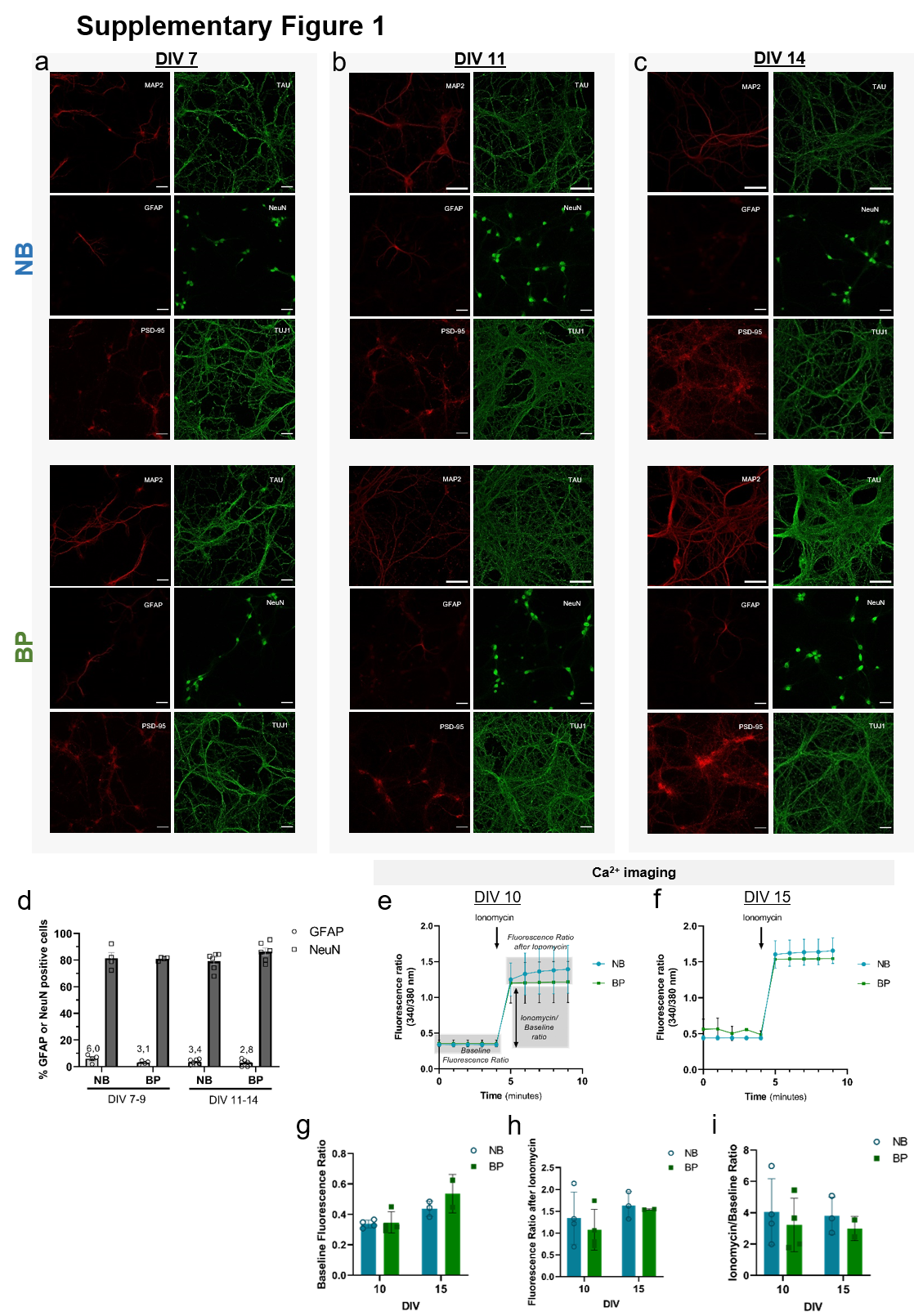


**Supplementary Figure 1: Denser neuronal networks in mouse neurons maintained in BP medium supported by immunofluorescence**

Immunofluorescence images of neurons maintained in NB or BP media collected at DIV7 (**a**), DIV11 (**b**) or DIV14 (**c**) and stained for neuronal markers: MAP2, TAU, NeuN, PSD-95 and TUJ1, and astrocytic marker: GFAP. All scale bars represent 40µm. (n=2-3 biological replicates). Quantification of the % of GFAP (white bars) and NeuN (grey bars) positive cells in primary neurons maintained in NB or BP at DIVs 7-9 and DIVs 11-14 (d). Time course of Ca^2+^-dependent fluorescence ratio (F340/F380) following 2 µM ionomycin (Ca^2+^ ionophore) treatment in neurons maintained at DIV10 (e) and DIV15 (f). From these traces, comparison of baseline Ca^2+^ fluorescence ratio is represented in (g); comparison of Ca2+ fluorescence ratio after ionomycin (h) and the comparison of ratio response after ionomycin and baseline fluorescence is depicted in (i). All graphs show means ± S.E.M., (n=2-4 biological replicates).


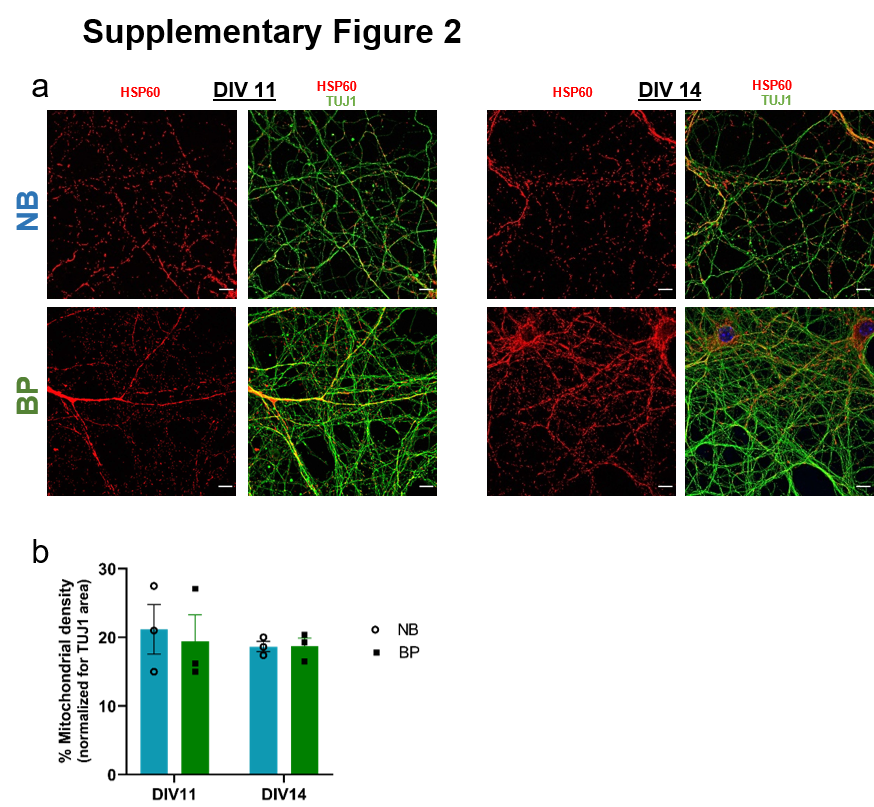


**Supplementary Figure 2: Mitochondrial density is similar between mouse neurons maintained in NB and BP medium**

Immunofluorescence images of neurons maintained in NB or BP media collected at DIV11 and DIV14 (**a**) and stained for neuronal marker, TUJ1 (in green), and mitochondrial marker, HSP60 (in red). Quantification (**b**) of the mitochondrial density (in percentage) in neurons maintained in NB and BP media at DIV11 and 14. All scale bars represent 10µm. (5-10 images were analysed from n=3 biological replicates)

**Refererences**

Barhoumi, R., Qian, Y., Burghardt, R. C., and Tiffany-Castiglioni, E. (2010). Image analysis of Ca2+ signals as a basis for neurotoxicity assays: Promises and challenges. *Neurotoxicol. Teratol.* 32, 16–24. doi: https://doi.org/10.1016/j.ntt.2009.06.002.

Ferreira, D. G., Temido-Ferreira, M., Miranda, H. V., Batalha, V. L., Coelho, J. E., Szegö, É. M., et al. (2017). α-synuclein interacts with PrP C to induce cognitive impairment through mGluR5 and NMDAR2B. *Nat. Neurosci.* 20, 1569–1579. doi: 10.1038/nn.4648.

González-Sánchez, P., del Arco, A., Esteban, J. A., and Satrústegui, J. (2017). Store-Operated Calcium Entry Is Required for mGluR-Dependent Long Term Depression in Cortical Neurons. *Front. Cell. Neurosci.* 11, 363. doi: 10.3389/fncel.2017.00363.

Hangen, E., Cordelières, F. P., Petersen, J. D., Choquet, D., and Coussen, F. (2018). Neuronal Activity and Intracellular Calcium Levels Regulate Intracellular Transport of Newly Synthesized AMPAR. *Cell Rep.* 24, 1001-1012.e3. doi: https://doi.org/10.1016/j.celrep.2018.06.095.

Jackson, T. C., Kotermanski, S. E., Jackson, E. K., and Kochanek, P. M. (2018). BrainPhys® increases neurofilament levels in CNS cultures, and facilitates investigation of axonal damage after a mechanical stretch-injury in vitro. *Exp. Neurol.* 300, 232–246. doi: https://doi.org/10.1016/j.expneurol.2017.11.013.

Knot, H. J., Laher, I., Sobie, E. A., Guatimosim, S., Gomez-Viquez, L., Hartmann, H., et al. (2005). Twenty years of calcium imaging: cell physiology to dye for. *Mol. Interv.* 5, 112–127. doi: 10.1124/mi.5.2.8.

Park, J., Lee, S. B., Lee, S., Kim, Y., Song, S., Kim, S., et al. (2006). Mitochondrial dysfunction in Drosophila PINK1 mutants is complemented by parkin. *Nature* 441, 1157–1161. doi: 10.1038/nature04788.
